# Hierarchical Novelty Detection for Traffic Sign Recognition

**DOI:** 10.3390/s22124389

**Published:** 2022-06-10

**Authors:** Idoia Ruiz, Joan Serrat

**Affiliations:** Computer Vision Center and Computer Science Department, Universitat Autònoma de Barcelona, 08193 Bellaterra, Spain; joans@cvc.uab.cat

**Keywords:** novelty detection, hierarchical classification, deep learning, traffic sign recognition, autonomous driving, computer vision

## Abstract

Recent works have made significant progress in novelty detection, i.e., the problem of detecting samples of novel classes, never seen during training, while classifying those that belong to known classes. However, the only information this task provides about novel samples is that they are unknown. In this work, we leverage hierarchical taxonomies of classes to provide informative outputs for samples of novel classes. We predict their closest class in the taxonomy, i.e., its parent class. We address this problem, known as hierarchical novelty detection, by proposing a novel loss, namely Hierarchical Cosine Loss that is designed to learn class *prototypes* along with an embedding of discriminative features consistent with the taxonomy. We apply it to traffic sign recognition, where we predict the parent class semantics for new types of traffic signs. Our model beats state-of-the art approaches on two large scale traffic sign benchmarks, Mapillary Traffic Sign Dataset (MTSD) and Tsinghua-Tencent 100K (TT100K), and performs similarly on natural images benchmarks (AWA2, CUB). For TT100K and MTSD, our approach is able to detect novel samples at the correct nodes of the hierarchy with 81% and 36% of accuracy, respectively, at 80% known class accuracy.

## 1. Introduction

**Figure 1:**
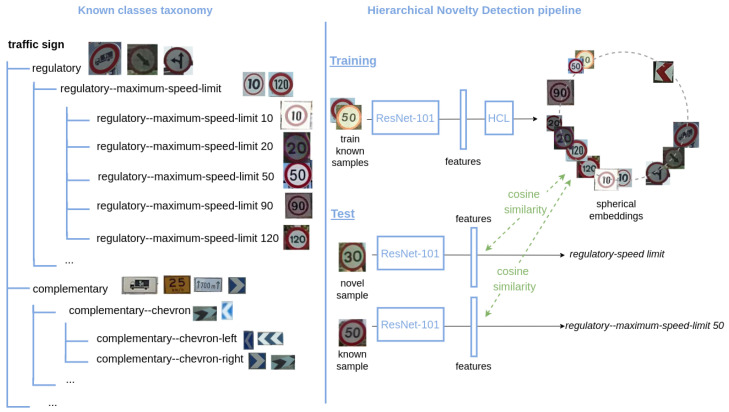
Example of hierarchical novelty detection on traffic sign recognition. Our system is trained to recognize speed limit traffic signs of 10, 20, 50, 90, and 120. When it is fed an image of a speed limit traffic sign of 30, it should predict that it belongs to a novel class (never seen during training), but also that it is a *speed limit* traffic sign, placing correctly the novel sample in the hierarchical taxonomy of known classes.

Deep neural networks have demonstrated to achieve outstanding performance on image classification. However, the problem of detecting samples that do not belong to any class known by the model, i.e., novelty detection, remains unsolved. Two challenges of this task are that, first, classification networks trained by cross-entropy tend to be overconfident about their predictions, meaning they will assign a known class to any input fed to the network with very high confidence. The second difficulty is that, by definition, there is no training data for what is *novel*. There have been some efforts in addressing such problem [1,2,3,4], but the binary output of these approaches only determines whether the sample belongs to a known class or is unknown. A desirable feature of classifiers would be, besides providing a novel/known decision, to produce an approximate prediction of the novel class by taking advantage of the knowledge of the already learned classes. In particular, we go beyond vanilla novelty detection and study how to perform such enhanced novelty detection under the framework of a hierarchical taxonomy of classes. This problem is known as hierarchical novelty detection [5]. It aims at correctly classifying samples of known classes, while also allocating the novel samples to the most suitable node of the hierarchy, i.e., their parent class. Figure 1 illustrates a simplified example. Let us assume a model trained on traffic sign recognition that has learned to only recognize speed limit traffic signs of 10, 20, 50, 90, and 120. If the system is fed a sample image of a 30-speed limit, it then should predict that this sample belongs to a novel class, and more precisely that it is a *speed limit* sign.

This problem has been traditionally studied as two independent tasks in the literature, i.e., novelty detection and hierarchical classification. Solving the joint task, however, has the advantage that novelty detection can benefit from the hierarchical taxonomy of classes. In academia, experiments are often restricted to certain sets of classes that compose the datasets. These data are limited and cannot comprise all the possible classes and variability of samples a real-life application faces. These experiments, moreover, are based on a closed-world assumption [6,7], i.e., systems consider the only existing classes are those seen at training time. Novelty detection, instead, necessarily considers an open world setting. By leveraging the hierarchical taxonomy of the known classes, we show it is possible to produce approximate predictions even for unknown samples, by classifying them to the closest concept in such semantic taxonomy.

In this work, we propose to solve the problem of hierarchical novelty detection by introducing a novel loss function, i.e., Hierarchical Cosine Loss (HCL), which learns an embedding of discriminative features that is consistent with the taxonomy class relationships by encoding taxonomy-based constraints. In this embedding, every known class that corresponds to either a parent or leaf node is represented by a *prototype*. *Prototypes* enable the classification of any kind of sample, including novel ones. HCL is based on a normalized version of the softmax loss reformulated from a cosine perspective. It optimizes the cosine similarity at training time between features and corresponding class *prototypes*. Consistently, we perform the novelty decision at test time by using the same metric. By mapping the sample into the embedding space, our approach assigns the sample features to the *prototype* with the highest cosine similarity.

To the best of our knowledge, there is only a previous work [5] that has approached this problem. The authors instead employ confidence calibrated classifiers [3] to overcome the difficulty of the overconfidence of models trained with standard softmax. Similar approaches to ours [8] have been proved to increase the performance on the face recognition task in comparison to the standard softmax formulation that the methods in [5] apply. Whereas the mathematical background is similar, there is a subtle conceptual difference among both formulations. While standard cross-entropy answers the question *What is this sample?* ours tries to find the response to *What does this sample look like most?* The latter setting seems to be more appropriate to classify unknown samples by finding the most similar known class.

Our solution can be a powerful tool for practical applications. For instance, a potential application is to ease annotation procedures that could be semi-automated by providing the closest known class even for novel samples. In addition, it could be a first step towards class incremental learning [2,9], where one could extend the model with the newly learned classes. As a concrete application, autonomous driving technologies can benefit from it, e.g., by detecting new object categories automatically in a navigation system and suggesting the most similar known class. The aforementioned class incremental setting could also be used to build adaptive models to the challenging changing environment that autonomous driving systems face. We specifically address the traffic sign recognition problem [10,11,12,13]. This task is of special interest because, in the case of traffic signs, the semantic taxonomy is strongly related to the visual appearance. The categories are human-built so that the meaning is intended to be visually represented. Therefore, one could build an adaptive traffic sign detector that is able to infer the meaning, at least partially, of the detected novel signs.

In summary, the contributions of this work are the following:

- A hierarchical novelty detection framework that is able to detect novel samples that belong to classes not seen during training, also placing them at the correct node of the taxonomy, i.e., predict the parent class. For this purpose, we introduce a novel loss function, i.e., Hierarchical Cosine Loss, which incorporates hierarchical constraints and optimizes the cosine similarity as the confidence metric, differently from most of current approaches that are based on class probabilities.
- A specific application to traffic sign recognition. We introduce the taxonomies and appropriate splits for two large scale traffic signs datasets, Mapillary Traffic Sign Dataset (MTSD) and Tsinghua-Tencent 100K (TT100K).
- We show that HCL significantly outperforms state-of-the-art approaches on these traffic sign benchmarks. For TT100K and MTSD, our method is able to detect novel samples from unknown classes at the correct nodes of the hierarchy with 75% and 24% accuracy when it correctly classifies known classes with 90% accuracy, respectively. It also reaches 81% and 36% novel accuracy at 80% known accuracy for TT100K and MTSD, respectively. Additionally, on the natural images datasets AWA2 and CUB, it achieves equivalent performance to state-of-the-art models.
- A new hierarchical novelty detection metric, i.e., the average error distance ${\bar{d}}_{h}$, to evaluate the errors produced under a hierarchical setting. It measures how far in the hierarchy we predict novel classes from the correct node.
- An ablation study that analyzes the individual performance of the HCL terms, discussing their benefits and drawbacks.

## 2. Related Work

### 2.1. Novelty Detection

Broadly, novelty detection belongs to the field of study of out-of-distribution detection [1,2,14,15], that consists of identifying samples that do not belong to the distribution of the training data (in-distribution). More specifically, novelty detection aims to classify known classes while detecting *novel* samples that correspond to classes never seen during training. For instance, the authors of [1] address out-of-distribution detection by proposing a metric learning based approach. They distinguish among *novelties* and *anomalies* depending on the resemblance w.r.t. the in-distribution data. Similarly to us, they apply it to traffic sign recognition. However, they only provide a binary output that classifies a sample into either a known class or a generic class of *novelty*. Our approach instead provides information about what kind of *novelty* it is, by predicting its parent class as the expected output. In a different direction, the work in [2] considers both out of distribution detection and adversarial attacks, as both problems consist of detecting abnormal samples. They propose a Gaussian discriminant analysis resulting in a confidence score based on the Mahalanobis distance. Moreover, they apply their approach into a class incremental setting framework, showing they are able to incorporate new classes without retraining the models.

There are no works other than [5] directly addressing hierarchical novelty detection. The reason is probably that it is a concrete and complex task that merges two problems traditionally studied separately, i.e., hierarchical classification and novelty detection. The authors of [5] introduced the problem for the first time and proposed two different models. The first *Top-Down* model trains confidence-calibrated classifiers [3], which, besides training the standard cross-entropy loss, minimize the KL divergence of the probability vector w.r.t. the uniform distribution. At test time, it makes top-down decisions so that at each node it measures the KL divergence to evaluate whether the classifier is confident about the prediction, which determines if the sample is novel or known when compared to a threshold. The second *Flatten* model trains the standard cross-entropy loss considering all classes, i.e., both leaf and super classes, and performs the decision ignoring the taxonomy. Additionally, they show the hierarchical embeddings can be employed to improve the performance on generalized zero shot learning. Both their proposed approaches employ the standard softmax objective and base their training and decision on class probabilities. Differently to them, we train our embeddings by optimizing the cosine similarity instead of the inner product and perform the novelty decision based on this similarity metric. Furthermore, our approach learns an embedding of discriminative features that is consistent with the taxonomy class relationships.

Nevertheless, there exist some problems that are conceptually similar. One of the closest problems is zero-shot learning (ZSL) [16,17,18,19], where the goal is to classify samples of classes not seen during training. The base idea of hierarchical novelty detection, i.e., to use the knowledge of the known classes to recognize the novel ones, is shared with ZSL. It, however, requires additional information about the known classes to be given, in the form of attributes or text description transformed into embeddings, while hierarchical novelty detection only relies on the class taxonomy.

### 2.2. Hierarchical Classification

Considering hierarchical class taxonomies in the classification problem has been widely studied in the literature [20,21,22,23,24]. The problem of hierarchical novelty detection actually comprises hierarchical classification of the known classes. In [20], the authors take advantage from hierarchical taxonomies of classes for error measurement. They propose two methods based on the cross-entropy loss that aim to minimize the asymmetric cost of the errors produced. Their error evaluation employs the height of the lowest common ancestor (LCA) among the predicted and the ground-truth classes in the taxonomy tree. This is similar to the metric we propose for the task of hierarchical novelty detection in Section 5.2.1, the average error distance ${\bar{d}}_{h}$. Differently, we use the distance in the tree between both classes that corresponds to the sum of distances from both the predicted and ground truth classes to their LCA. More recently, a *prototypical network* is introduced in [21] that is supervised by employing a cost matrix that encodes hierarchical relationships among classes, then penalizing large hierarchical errors. It is conceptually similar to our proposed loss in that they also incorporate hierarchical constraints to learn an optimal embedding. In addition, they consider the *Average Hierarchical Cost* as a metric to evaluate classification errors, which matches the definition of our average error distance ${\bar{d}}_{h}$ metric, but in the context of hierarchical classification.

Another related problem is long-tailed recognition that consists of correctly classifying classes from which many of them are underrepresented in the training data. This often matches a real-life scenario, where having balanced data for all the classes is unlikely. The obvious differences are that their classes are highly imbalanced but at least one sample per class is seen during training, and they do not need to make a novel/known class decision. Some works employ class hierarchies in their solution. For instance, the authors of [25] propose to solve this problem under a hierarchical class taxonomy framework, then providing from coarse to fine-grained predictions according to the confidence. This enables the models to reject classification at different levels. More recently, the authors of [26] transforms the problem into a hierarchical classification one by building a tree whose levels correspond to different degrees of difficulty according to how imbalanced the data are, then transferring the knowledge across levels.

### 2.3. Cosine Losses

There exist diverse works that, similarly to us, propose loss functions based on modifying the softmax loss from a cosine perspective to improve its performance. The softmax loss, in this context, refers to a cross entropy loss preceded by a softmax activation and a fully connected layer. These works are commonly applied to the face recognition task, where learning discriminative features is essential to distinguish identities. They also benefit from this loss formulation because they use the cosine similarity at test time.

The first work that opened this line of research was [27], where, based on the softmax loss, the proposed L-softmax loss included a new angular margin hyperparameter that acts on the class decision boundaries to enforce inter-class variance and then push the discriminative power of the features. SphereFace [28] normalized the weights of the last fully-connected layer on the L-softmax loss, making them lie on a hyphersphere. A normalized version of the softmax loss was introduced in [8], where they normalized both features and class weights so that the only variable to be optimized is the cosine of the angle between them. Later, the authors of [29] added a margin parameter to it to increase the discriminative power of features. This margin separates the decision boundary between classes in the embedding space, at the cost of introducing a new hyperparameter. In our work instead, to learn discriminative features under a hierarchical setting, we propose additional terms to the loss that encode hierarchy-based constraints, being consistent with the problem we aim to solve. Similarly to [29], the authors of [30] also introduced a margin hyperparameter but applied on the angle. Finally, in an effort to improve the aforementioned methods, AdaCos [31] proposes a hyperparameter-free approach, leveraging a dynamically adaptive scale parameter that is adjusted automatically. Simultaneously, RegularFace [32] proposed an *exclusive regularization* term to the loss to further push inter-class discriminability by optimizing angular distance among classes.

## 3. Hierarchical Novelty Detection

In this section, we first describe the setting of the hierarchical novelty detection problem in Section 3.1 and then introduce our proposed Hierarchical Cosine Loss in Section 3.2.

### 3.1. Class Taxonomy

In hierarchical novelty detection, the classes are organized by a hierarchy of known classes that is built based on their semantics. The resulting taxonomies of the datasets considered in this work are trees, where all nodes have at least two children classes and a single parent. As an example, Figure 1 shows a subset of the taxonomy of MTSD. The dataset is split into two sets of disjoint classes: *known* and *novel*. *Known* classes are used during training to learn an embedding, while *novel* classes are not included in the hierarchy; they are never seen during training and our goal is to predict the correct parent (known) class for the novel samples at test time.

Datasets provide samples for known leaf classes. However, our approach also needs sets of samples that represent the parent classes. To this end, we employ a relabeling strategy as in [5]. We select a percentage of the samples of the leaf classes to be relabeled as their parent class. We refer to this percentage as the relabeling rate $r_{rate}$. This procedure is recursively repeated in a bottom-up manner from the bottom nodes to their parents until we reach the root and all the nodes are assigned samples. The subset of samples is chosen randomly and is different for each epoch.

### 3.2. Hierarchical Cosine Loss

We introduce the Hierarchical Cosine Loss (HCL) in order to learn an embedding for the known classes. HCL comprises a layer of learnable parameters that corresponds to a fully connected layer with no bias. The HCL layer is appended after the feature layer of a ResNet-101 backbone, which serves as a feature extractor. Our loss, HCL, is composed by a set of terms that enforce learning discriminative features, leveraging the class hierarchy. It is defined as follows:

(1) $$\mathrm{HCL}=\lambda_{NS}L_{NS}+\lambda_{HC}L_{HC}+\lambda_{CT}L_{CT}+\lambda_{HT}L_{HT},$$

where $L_{NS}$, $L_{HC}$, $L_{CT}$, and $L_{HT}$ stand for Normalized Softmax, Hierarchical Centers, C-triplet, and Hierarchical Triplet loss, respectively, and $\lambda_{NS}$, $\lambda_{HC}$, $\lambda_{CT}$, and $\lambda_{HT}$ are their regularization parameters.

**Normalized Softmax Loss**$L_{NS}$. A reformulation of the softmax loss was introduced in [8], consisting of applying normalization on both the weights from the last fully-connected layer, whose bias is set to 0, and the feature vectors. This results in optimizing the cosine similarity instead of the inner product. We refer to this loss as Normalized Softmax Loss (NSL) $L_{NS}$. NSL is defined as

(2) $$L_{NS}=\frac{1}{N}\sum_{i}-log\frac{e^{s\cos(\theta_{y_{i},i})}}{\sum_{j}e^{s\cos(\theta_{j,i})}},$$

where $y_{i}$ is the ground truth label of the *i*-th sample, *N* is the number of samples and $\theta_{j,i}$ is the angle between $W_{j}$ and $x_{i}$, with $W_{j}$ being a weight vector of the fully-connected layer for the *j*-th class and $x_{i}$ the feature vector of the *i*-th sample. A weight vector $W_{j}$ can be interpreted as a representative vector of the *j*-th class, and we refer to it as a class *prototype*. For a class whose features are properly separated in the embedding space, its *prototype* would correspond to the mean of the features. By applying $L_{2}$ normalization, we fix $∥W_{j}∥\;=1$ and ∥x∥ =s. This results in optimizing only the cosine of the angle, as the norms will not contribute to the loss. After normalization, the feature vectors lie on a hypersphere, where the scaling parameter *s* controls its radius and the resulting features are separable in the angular space, reducing intra-class angular variability and pushing inter-class variance within the hypersphere. This consequently enforces removing radial variations.

**C-Triplet loss**$L_{CT}$. This re-formulation of the softmax loss can also be translated into the Contrastive or Triplet losses, which inspired us to propose the following loss terms that incorporate hierarchical constraints. In [8], the authors introduce the *C-triplet* loss $L_{T^{\prime }}$ as the modified version of the triplet loss that is defined as follows:

(3) $$L_{T^{\prime }}=\max(0,m+∥{\tilde{x}}_{i}-W_{j}{∥}_{2}^{2}-∥{\tilde{x}}_{i}-W_{k}{∥}_{2}^{2}),\;\forall y_{i}=j,y_{i}\neq k,$$

where $\tilde{x}=\frac{x}{s}$ and *m* is a margin parameter. Note that both $\tilde{x}$ and $W_{j}$ are normalized, then we could re-formulate it in terms of the cosine similarity. Considering that $∥{\tilde{x}}_{i}-W_{j}{∥}_{2}^{2}\;=2-2W_{j}^{T}{\tilde{x}}_{i}$ and $W_{j}^{T}{\tilde{x}}_{i}=\cos\theta_{j,i}$, $L_{T^{\prime }}$ can also be defined as

(4) $$L_{T^{\prime }}=\max\left(0,m+2\cos\theta_{k,i}-2\cos\theta_{j,i}\right),\;\forall y_{i}=j,y_{i}\neq k.$$

Then, considering pairs of different classes i,j, we define our C-triplet loss term $L_{CT}$ as

(5) $$L_{CT_{i}}=\max\left(0,\cos\theta_{j,i}-\cos\theta_{i,i}+m_{CT}\right),\;\forall y_{i}\neq j.$$

where the margin parameter $m_{CT}$ is set to zero in all our experiments for simplicity. This term is intended to increase the discriminative power of the features, increasing the inter-class variance. It encodes that the features of a class should be closer to their class than to other class centers, i.e., the cosine similarity is higher among the features of a class $x_{i}$ and its *prototype* $W_{y_{i}}$ than to the *prototypes* of different classes $W_{j}|y_{i}\neq j$.

**Figure 2:**
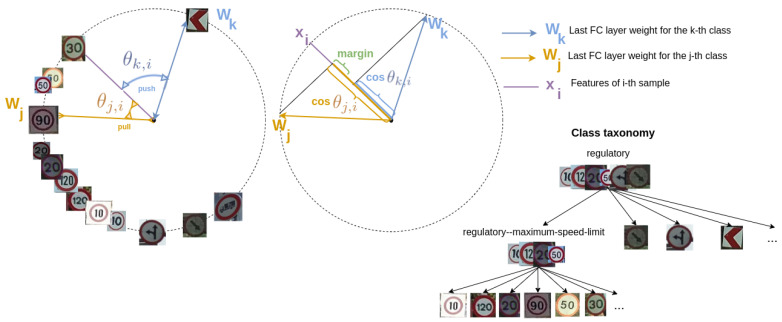
Interpretation of Hierarchical Triplet loss term $L_{HT}$. Samples are forced to be closer to *prototypes* of classes that are closer in the taxonomy. In this example, the anchor sample is a speed limit sign of 30, while the positive class is the speed limit sign of 90, which is closer in the taxonomy than the direction sign class that is the negative class of the triplet.

**Hierarchical Triplet loss**$L_{HT}$. To further enforce discriminative features based on the hierarchical relationships, we propose the Hierarchical Triplet term $L_{HT}$ that is defined as

(6) $$L_{HT_{i}}=\max\left(0,\cos\theta_{k,i}-\cos\theta_{j,i}+m_{HT}\right),\;\forall i,j,k|y_{i}\neq j\neq k,\;d_{h}\left(y_{i},j\right)<d_{h}\left(y_{i},k\right),$$

where $d_{h}$ is the hierarchical distance between two nodes in the taxonomy, and we refer the reader to Section 5.2.1 for more details on this distance. $m_{HT}$ is a margin parameter and is set to zero in all our experiments. The purpose of this term is that features of a class should be closer to the *prototypes* of those classes that are closer in the taxonomy. For instance, a *speed limit* traffic sign class will be closer to any other *speed limit* sign than to any *direction* traffic sign. Figure 2 illustrates an example. The effect of this term is then to distribute the features in the hypersphere according to the taxonomical relationships.

**Hierarchical Centers loss**$L_{HC}$. Similarly to the Hierarchical Triplets term $L_{HT}$, the Hierarchical Centers loss $L_{HC}$ aims to increase the separation in the angular space of the class *prototypes* $W_{j}$ based on the hierarchical relationships between classes. The difference is that, instead of being applied to the distance among features and *prototypes*, it only affects the class *prototypes* $W_{j}$. Thus, this term enforces a higher similarity among class *prototypes* that are closer in the taxonomy. $L_{HC}$ is defined as

(7) $$L_{C_{i}}=\max\left(0,\cos\phi_{y_{i},k}-\cos\phi_{y_{i},j}+m_{C}\right),\;\forall i,j,k|y_{i}\neq j\neq k,\;d_{h}\left(y_{i},j\right)<d_{h}\left(y_{i},k\right),$$

where $\phi_{y_{i},j}$ is the angle between $W_{y_{i}}$ and $W_{j}$. $m_{C}$ is a margin parameter and is set to 0.05 in all our experiments.

### 3.3. Inference

By training HCL for both known leaf and super classes, we learn the set of class *prototypes*, i.e., class weights $W_{j}$ from the last fully connected layer that identifies all the known classes. These, at test time, can be compared against the features of the test samples to perform classification.

At inference time, for every test sample, we compute the cosine similarity between its features and all the class *prototypes* $W_{j}$. These features are extracted from the ResNet-101 model as we do at training time. We add an offset to the cosine similarities of the super classes, which controls the trade-off between known and novel class accuracies. Its value can be varied within a range to select the desired working point. This is needed to compute the metrics detailed in Section 5.2. The test samples are finally classified to the class whose *prototype* $W_{j}$ has the highest similarity w.r.t. their features, after applying the offset. Then, if a sample is assigned a leaf class, it means it corresponds to a sample of this known leaf class, while if the sample is assigned a super class, it is considered as a novelty under this parent class. For instance, a sample classified as a *regulatory* traffic sign is a sign of an unknown class of type *regulatory* that in the taxonomy would be a child class of *regulatory*.

Note that, differently from other hierarchical classification methods [5], we do not follow a top-down strategy. This avoids top-down error aggregation that happens when the prediction at the top-most levels is wrong, and is magnified with complex and deep taxonomies. Therefore, we do the classification at inference time not considering any class taxonomy, all the classes being equally probable.

## 4. Datasets

We consider two kinds of datasets to assess the performance of our approach. First, to compare it against the state-of-the-art methods of hierarchical novelty detection, we employ the evaluation setting proposed in [5] as well as the datasets, CUB [33] and AWA2 [16]. Additionally, because we aim to apply our method on a traffic sign recognition framework, we choose two large scale traffic sign benchmarks: Tsinghua-Tencent 100K (TT100K) [34] and Mapillary Traffic Sign Dataset (MTSD) [35].

The original classes of these datasets are split into known and novel. Those that are known correspond to the leaf classes of the hierarchy. Among the samples of the known leaf classes, we build train, validation, and test splits. Train samples are used to train the model, validation for hyperparameter optimization, and test samples are used to evaluate the classification accuracy on the known classes. The details on how we make the splits are detailed in the following sections for each dataset. The data to reproduce our experiments are available in [36].

A dataset is more challenging as it has a larger number of samples, categories, and has a more complex taxonomy of classes [37]. Table 1 contains these data for the datasets evaluated in this paper. Note this information corresponds to the samples used in our experiments, where we have discarded some of the samples, and may differ from original benchmark statistics. Finally, the class taxonomies for these benchmarks are shown graphically in Appendix B.

### 4.1. Tsinghua-Tencent 100K (TT100K)

The Tsinghua-Tencent 100K [34] dataset is one of the first large scale traffic sign benchmarks. It contains samples under different illuminance and weather conditions, extracted from real-life street view panoramas. In our experiments, we have used the cropped images of traffic signs. A difficulty of this dataset is that it is highly imbalanced. It is built from real-life images, where different traffic signs do not appear with the same frequency. Only 45 classes out of 221 have more than 100 examples, while the largest class has 2819 samples.

The criteria to decide which classes belong to the novel split are based on the number of samples per class. We first discard the classes with less than 10 samples to avoid errors. From the remaining classes, we take 20% of those least populated as novel, regardless of their position in the taxonomy. We think this split is the one that best simulates the data in a real-world application, i.e., for a novel class, the goal is to correctly classify its samples in the taxonomy, not needing many of them, while known classes should be properly learnt from a larger number of samples. The most logical option is therefore to select the classes with fewer samples as novel. To build the train/test splits, for each known class, we keep 20% of the samples for tests and, within the remaining samples, 20% are used for validation.

Since this dataset has not been previously used for hierarchical novelty detection, we have built a taxonomy based on class semantics, e.g., for traffic signs of *prohibition limit of 20*, they should have a parent class that comprises *prohibition limit* signs at other speeds, while this class should have a parent class that comprises any kind of *prohibition* sign as well. A visual representation of the built taxonomy is shown in Appendix B.

### 4.2. Mapillary Traffic Sign Dataset (MTSD)

Recently, the Mapillary Traffic Sign Dataset has been introduced in [35]. It is the largest and most diverse traffic sign benchmark up to date. While TT100K contains only standard circular and triangular shaped signs, MTSD also includes direction, information or highway signs. Moreover, the images have been captured by multiple different camera devices all over the world. The benchmark provides fully and partially annotated traffic signs, although our experiments are restricted to only the fully annotated samples. Similarly to TT100K, MTSD is imbalanced, despite having a larger number of samples per class.

**Figure 3:**
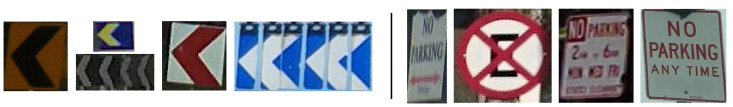
Two examples of samples of MTSD that are distinguished as disjoint classes in the original benchmark because they share the same semantics but have a different appearance. Aiming at classifying according to semantics, we merge them as single classes. In the left case, we merge five classes into one (*complementary–chevron-left*) and, in the right case, we merge four classes into one (*regulatory–no-parking*).

The original class taxonomy of MTSD distinguishes as independent classes those that contain templates with the same semantics and similar appearance. However, we consider semantic based taxonomies, i.e., our application intends to classify the samples according to their meaning and not their appearance. For consistency, we choose to merge different groups of templates that share the same semantics but are different in terms of appearance, into a single class, as shown in Figure 3. This increases the intra-class variability, but in exchange simplifies the taxonomy we would have if we distinguished these classes. From 313 classes in the original taxonomy, after merging those classes with the same semantics, the resulting taxonomy has 203 leaf classes. Among these, 74 classes have less than 100 samples, while the largest class has 2775 samples.

To build the hierarchical taxonomy, we create super classes that encompass the traffic sign categories provided in MTSD that share similar semantics, e.g., the classes *regulatory–no-left-turn* and *regulatory–no-right-turn* have a parent class *regulatory–no-turn* that at the same time will have a *regulatory* parent class that comprises all the *regulatory* signs. Note that, due to the different composition of classes of TT100K and MTSD, they do not share a unified traffic sign taxonomy. There is no universal traffic sign taxonomy, to the best of our knowledge. It would be an interesting objective to explore in future work or a practical application, however.

Finally, we make the novel/known and train/test/validation splits by employing the same criteria and percentages as for TT100K.

### 4.3. AWA2, CUB

We employ the taxonomies of AWA2 and CUB provided in [5], which are built from the WordNet hierarchy by using its hypernym–hyponym relationships. Visual representations of the resulting hierarchies can be found in Appendix B. An interesting aspect of these taxonomies, differently to traffic signs, is that they obey to semantic hierarchical categories such as: placentalmammal→carnivore→canine→dog→shepherddog, where *carnivore* contains children classes as diverse as *bear* or *feline*. These high-level categories have no clear common features based on visual appearance. It is probably harder to learn how a *carnivore* looks than how a *prohibition* sign looks, since the concept is not reflected in the appearance but in deeper knowledge about what being a *carnivore* involves. These broad concepts are translated into a larger variability of samples under such conceptually high-level classes as well.

## 5. Evaluation

### 5.1. Experimental Setup

We compare our method against the state-of-the-art models proposed in [5]. They propose three models, from which we consider *TD+LOO* and *Relabel* for the sake of a fair comparison. *TD+LOO* is their best performing model, while *Relabel* uses the same relabel strategy as ours to assign samples to parent classes. We run the implementation of these models provided by the authors.

To train our model, we consider two settings depending on the experiment. The first one consists of using fixed precomputed features by freezing the weights of the ResNet-101 backbone, while training the HCL fully connected layer to learn the *prototypes* $W_{j}$ of the classes, as detailed in Section 3.2. In this setting, we train HCL but not the ResNet-101 backbone. In the second setting instead, we train jointly all the layers of ResNet-101 and HCL.

While carrying out the experiments, we noticed there was a moderate variability in the results even when using the same set of hyperparameters. Therefore, we repeat several times each experiment for a fixed set of hyperparameters. Instead of reporting the best-performing experiment from a set, the variability of the method is worth being analyzed. As we shall optimize our model to the validation set, a method whose performance is highly variable is not reliable because we do not know how it will perform on the test set.

All our experiments are run on a set of GeForce GTX 1080, using multiple devices (at most four) in parallel when necessary, depending on the batch size.

### 5.2. Metrics

In order to assess the performance of our method on hierarchical novelty detection, we consider the following metrics. For comparison against the state-of-the-art approach proposed in [5], we employ the AUC of the novel/known accuracy curve and the novel accuracy at a fixed known accuracy point. In their work, they select the point of 50% known accuracy as a reference. We use the average top-1 accuracy, so that a correct prediction is defined as follows: depending on the split. For known classes, their correct prediction is the ground truth label, while for novel classes, a correct prediction involves classifying it as the closest class in the taxonomy, i.e., its parent known class. The accuracy is averaged by the number of samples, independently of their label.

The novel/known accuracy curve is obtained by adding an offset to the similarity metrics of the potential novel classes, i.e., parent nodes. This offset value is varied so that we increase/decrease novel accuracy in detriment/favor of known accuracy, as both splits hold a trade-off relationship. The novel/known accuracy curve is built from a range of offset values that allows for exploring all the available accuracy ranges. Accordingly, the AUC value is independent from the offset, i.e., it is independent from the working point.

On traffic sign benchmarks, we additionally consider points of interest at higher known accuracy points. In this context, we are interested in a working point in which our system classifies correctly most of the known classes, while performing as best as possible on the unknown ones. For this reason, the metrics that are more relevant are those of higher known accuracy points. In particular, we report the novel accuracy at 70% and 80% known accuracies, although we are interested in the range of known accuracy over 70%. For this reason, the AUC value is not a highly representative metric in our analysis, as it corresponds to the area for the full range.

#### 5.2.1. Hierarchical Error Distance

The accuracy only evaluates if the prediction matches the correct label but does not provide a measurement of the errors made. Specially under a hierarchical setting, we find this metric to be insufficient. Two wrong predictions of different degree of importance are treated as equally wrong by the accuracy. For instance, if the true class of a sample is a *20 maximum speed limit regulatory* sign, predicting its class as a *10 speed limit regulatory* sign should be considered a smaller error than predicting it as a *chevron left complementary* sign. In fact, other works on hierarchical image classification [20,21] stress the importance of optimizing error based metrics besides accuracy.

As a complementary metric to the accuracy, we introduce the hierarchical average error distance ${\bar{d}}_{h}$. It corresponds to the distance between the predicted and the correct class in the taxonomy tree. For the *i*-th sample, the hierarchical error distance $d_{h}\left(p_{i},y_{i}\right)$ between the predicted class $p_{i}$ and its ground truth label $y_{i}$ is defined as the length of the shortest path in the tree that corresponds to the sum of distances from both classes $p_{i}$ and $y_{i}$ to their lowest common ancestor (LCA). The average error distance ${\bar{d}}_{h}$ is then defined as

(8) $${\bar{d}}_{h}=\frac{1}{N}\sum_{i}d_{h}\left(p_{i},y_{i}\right),$$

where *N* is the total number of samples. Note that this distance metric is not normalized by the height of the taxonomy tree, which affects its maximum value, e.g., in a taxonomy of five levels, the maximum error distance is 10, while, in a taxonomy of 2 levels, it is 4.

In our experiments, we report the hierarchical average error distance for the novel split only, to analyze its dependency w.r.t. the accuracy of the known split. This provides a measurement of the novelty detection error under such hierarchical setting.

## 6. Results and Discussion

Our experiments are divided into three parts. To evaluate the performance of our approach, HCL, in Section 6.1, we compare it to the state-of-the-art models in hierarchical novelty detection, i.e., *TD+LOO* [5] and *Relabel* [5]. In Section 6.1.1, we first consider the benchmarks where these models were originally evaluated, i.e., AWA2 and CUB. Then, in Section 6.1.2, we perform the evaluation on the target traffic signs benchmarks TT100K and MTSD. In the next sections, we provide a more exhaustive evaluation of HCL on TT100K and MTSD. We compare the performance of different training strategies in Section 6.2. Finally, in Section 6.3, we analyze the individual contribution of each of the terms of HCL.

### 6.1. Comparison to the State of the Art

#### 6.1.1. AWA2 and CUB

For these experiments, we train HCL, *TD+LOO* and *Relabel* on top of features extracted from a ResNet-101 model that is only trained on ImageNet. This is the setting the authors of [5] chose, in their case claiming speed reasons. We use the exact hyperparameters and setting indicated by the authors. For HCL, the hyperparameters are chosen by optimizing them to the validation set (see Appendix A for details on hyperparameters).

**Figure 4:**
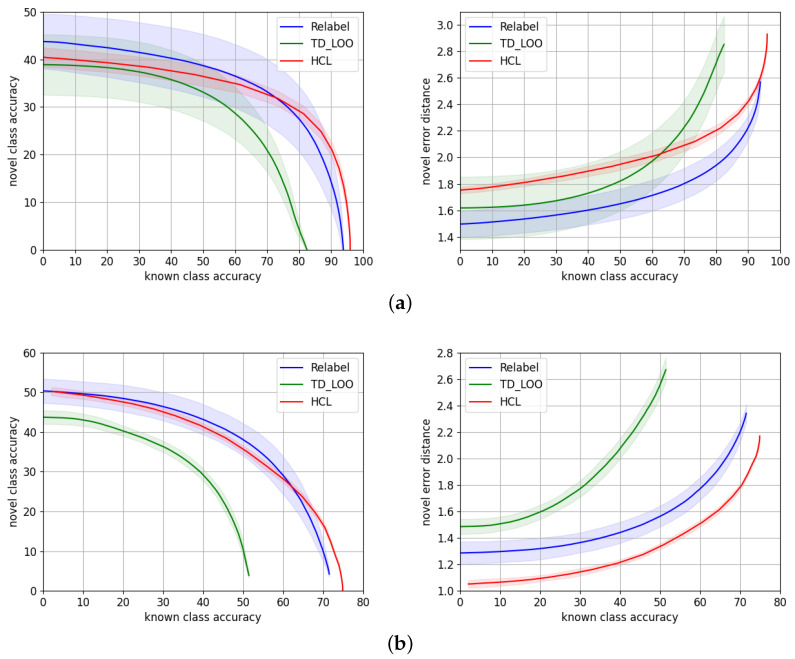
Novel/known accuracy trade-off and novel average hierarchical error distance over known accuracy, for HCL (red) and the state-of-the-art models *TD+LOO* [5] (green) and *Relabel* [5] (blue) for (**a**) AWA2 and (**b**) CUB.

We report in Table 2 the metrics introduced in Section 5.2 comparing the performance of our approach to *TD+LOO* and *Relabel*. The values of the metrics correspond to the average of 50 experiments, and we provide an error of ±2σ. Figure 4 shows the novel/known accuracy trade-off and the average hierarchical error distance on the novel split over the known split accuracy. The dark curves of the plots correspond to the average of the set of 50 repeated experiments, while the shaded area around illustrates ±2σ for each point.

**AWA2**. Considering only the mean of the experiments, *Relabel* [5] is superior in terms of accuracy in the range up to 70% known accuracy, while HCL performs better in the highest known accuracy range. This is the reason why *Relabel* obtains the highest AUC. However, if we take into account the variability of the methods, HCL and Relabel perform very similarly, i.e., their curves overlap except for the highest known accuracy range. Regarding the novel hierarchical error distance ${\bar{d}}_{h}$, *Relabel* [5] consistently makes smaller errors on the novel split.

**CUB**. In terms of accuracy, both *Relabel* and HCL perform similarly although the variability of *Relabel* is higher. *Relabel* performs better than the other variants up to ∼60% known accuracy while HCL is superior in the uppermost known accuracy range. HCL makes a consistent smaller average error through all the accuracy ranges.

Our results show HCL performs similarly to the state-of-the-art methods [5] on the natural images benchmarks, AWA2 and CUB. It shows a slightly higher novel accuracy at the highest known accuracy ranges, while the errors made by the model are smaller than *TD+LOO* and *Relabel* on CUB but higher on AWA2.

#### 6.1.2. TT100K and MTSD

Instead of using the features from a model trained on ImageNet as in the previous experiments, for TT100K and MTSD, we find it necessary to perform a fine-tuning of ResNet-101 using the cross-entropy loss. This is because traffic signs are a very specific kind of data, with a visual appearance different to ImageNet images. The comparison of performance when using features fine-tuned or not to the target dataset will be discussed later in Section 6.2.

The fine-tuning is performed by training ResNet-101 for 1000 epochs using a batch size of 140 and a learning rate of $1\times 10^{-4}$ with an Adam optimizer for both datasets. Once ResNet-101 is trained, we extract the features to train *TD+LOO*, *Relabel* and our model, as we did for AWA and CUB. It is also possible to train simultaneously the ResNet-101 backbone and HCL. However, we chose to do a separate fine-tuning to keep the setting proposed in [5] for the sake of a fair comparison. The fine-tuning was performed only once, while the experiments for HCL, *TD+LOO*, and *Relabel* were repeated 50 times with the set of best performing hyperparameters on the validation set. We refer the reader to Appendix A for details on hyperparameters.

**Figure 5:**
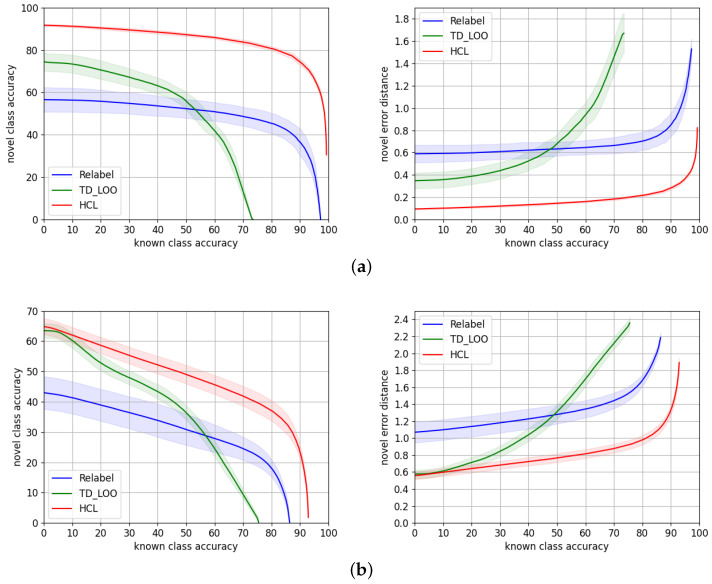
Novel/known accuracy trade-off and novel average hierarchical error distance over known accuracy, for HCL (red) and the state-of-the-art models TD+LOO (green) and Relabel (blue) for (**a**) TT100K and (**b**) MTSD.

We compare in Table 3 HCL to *TD+LOO* and *Relabel*. The reported metrics are the average value ±2σ from the set of 50 experiments. Figure 5 shows the novel/known accuracy trade-off and the average hierarchical error distance on the novel split over the known split accuracy.

On both datasets, HCL consistently outperforms *Relabel* [5] and *TD+LOO* [5] by a large margin through all the ranges of accuracy both in terms of accuracy and average novel hierarchical error distance. Our results suggest that our approach is more suitable for traffic signs’ datasets. A possible explanation is related to the taxonomy of these datasets. Both *TD+LOO* and *Relabel* solely rely on the cross-entropy loss, but HCL learns an embedding of discriminative features that could benefit from taxonomies related to the visual appearance of the classes, e.g., *prohibition* signs have common visual features, while *carnivore* images do not have an indistinguishable visual feature.

### 6.2. Training Strategies

We consider three different settings to train HCL. In particular, we train HCL on top of features that are extracted from ResNet-101 models that are previously trained, either on only ImageNet, or fine-tuned to the target dataset via the cross-entropy loss. The third setting we compare is when we train simultaneously HCL and the ResNet-101 backbone that is pretrained on ImageNet. We keep the fine-tuning procedure that is detailed in the previous Section 6.1.2.

Using the hyperparameters from Table A1, we repeat each new experiment for 10 times, as some training variants we compare in this section are time-consuming, and HCL was shown to be not so variable in previous results.

**Figure 6:**
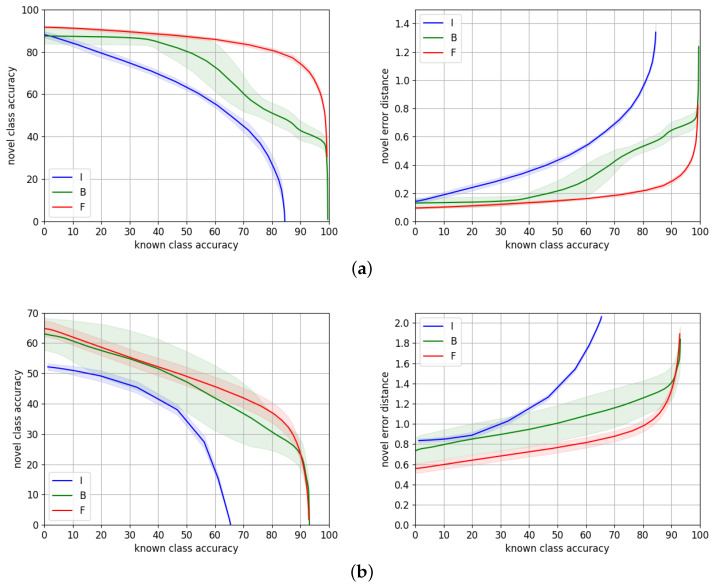
Novel/known accuracy trade-off and novel average hierarchical error distance over known accuracy, for different training strategies for HCL on (**a**) TT100K and (**b**) MTSD. I stands for ImageNet features, F for fine-tuned features, and B for training both the backbone and HCL simultaneously.

Table 4 reports the average metrics ±2σ for these three training strategies at 50%, 70%, and 80% known accuracy points, while the performance through the entire range is depicted in Figure 6.

The gap of performance on both datasets between using features from a network only trained on Imagenet, and features fine-tuned to the target dataset, justifies the need of performing such fine-tuning, especially because, in a traffic sign recognition application, we aim to maximize the novel accuracy at the highest known accuracy range.

On **TT100K**, training HCL on top of fine-tuned features works significantly better than training jointly HCL and the ResNet-101 backbone. This is probably because fine-tuning on TT100K is overfitting the dataset, which has few samples of a very specific kind of data (traffic signs). Then, training HCL from high quality features as a starting point is much easier than jointly learning suitable features along with proper class *prototypes* that are consistent with the taxonomy.

However, **MTSD** is a much larger dataset with a greater number of classes with higher inter and intra-class variability, as discussed in Section 4.2. The gap of performance in this case is therefore smaller. The accuracy curve overlaps for almost the entire range, although the gap in error distance is consistent. This means for a very similar number of correct predictions, and the errors of the wrong predictions are smaller when we use fine-tuned features, presumably because these fine-tuned features allow for doing a more precise classification of novel samples. The reason might be learning two objectives, i.e., class *prototypes* and suitable features, is a more challenging task than only learning the *prototypes* with a fixed set of features. This involves a less noisy signal to learn from, since features are not being updated during training. This effect that also occurred on TT100K at a smaller scale is magnified with larger datasets with high intra-class variability, as in this case.

As expected, the variability of the experiments when we train the backbone is higher than when we only train HCL, for both datasets. This is due to the additional variability introduced by training ResNet-101.

To conclude, our proposed approach, HCL, reaches its highest performance when trained from features fine-tuned to the target dataset. It is able to predict correctly 75% and 24% of novel samples, for TT100K and MTSD, respectively, when we predict known samples with 90% accuracy. It also predicts novel samples with an accuracy of 81% and 36% at 80% known accuracy for TT100K and MTSD, respectively.

### 6.3. Ablation Study of Hierarchical Cosine Loss

In order to analyze the individual contribution of the terms of HCL (Equation (1)), we conduct the following ablation study. We take as a baseline the contribution of only the Normalized Softmax loss (NSL) $L_{NS}$, then adding the contribution of the remaining terms that will be finally compared to an experiment in which all the terms contribute to the training. The latter is the best performing HCL experiment, according to the validation set. We train the terms of HCL over a set of constant features, fine-tuned to the target dataset. These fine-tuned features correspond to those used in the previous experiments. Note that this independent training of HCL allows for isolating the effect of the loss. Otherwise, training jointly the backbone and HCL would introduce a variability that would mask the actual variation of the individual loss terms.

In Table 5, we assess the individual performance of the different terms of HCL, defined in Section 3.2, on features fine-tuned to MTSD or TT100K. For each dataset, the first row shows, as a baseline, the metrics when we set the HCL regularization parameters to $\left\{\lambda_{NS},\lambda_{HC},\lambda_{CT},\lambda_{HT}\right\}=\left\{1,0,0,0\right\}$, i.e., we train using only the NSL $L_{NS}$. In the experiments of the next rows, we keep $\lambda_{NS}=1$ and add the different terms on each experiment, e.g., the second row corresponds to $\left\{\lambda_{NS},\lambda_{HC},\lambda_{CT},\lambda_{HT}\right\}=\left\{1,10,0,0\right\}$ where only the NSL $L_{NS}$ and Hierarchical Centers term $L_{HC}$ are contributing to the training. Similarly, the third row corresponds to the experiments where we use only the $L_{NS}$ and $L_{CT}$ terms with regularization parameters $\left\{\lambda_{NS},\lambda_{HC},\lambda_{CT},\lambda_{HT}\right\}=\left\{1,0,1,0\right\}$, and in the fourth row we train using $L_{NS}$ and $L_{HT}$ regularized by $\left\{\lambda_{NS},\lambda_{HC},\lambda_{CT},\lambda_{HT}\right\}=\left\{1,0,0,0.1\right\}$. The last row, where $\left\{\lambda_{NS},\lambda_{HC},\lambda_{CT},\lambda_{HT}\right\}=\left\{1,10,1,0.1\right\}$, reports the performance of the full version of HCL, including all the terms. Despite the NSL weight $\lambda_{NS}$ always being set to 1, we made sure its contribution to the loss was not leading the training, i.e., the loss that is being analyzed individually at each case is not being neglected and actually contributes to the training. That is, we made sure that the weights applied to the individual terms were appropriate to show the individual effect of the loss terms. Each training variant has been repeated 10 times, and we report an error of 2σ on Table 5.

For **TT100K**, the first experiment, in which we only use the NSL $L_{NS}$, obtains the best average metrics among the compared variants. However, the difference of performance is very small. In fact, if we take into account the variability of the experiments, we could consider all the variants to perform similarly. The cause of this result might be that the performance on this dataset is so good that it reaches a limit that is hard to surpass. Making small modifications on the loss is not translated into a significant change in performance. In this scenario, making even very small improvements is not straightforward, and it would probably mean it is overfitting the dataset.

The results on **MTSD** are more enlightening; it is a more challenging dataset, closer to a real-life scenario. Using the different terms of HCL always improves the average novel accuracy at 70% and 80% known accuracies w.r.t. the NSL baseline. The variant that performs best on these metrics is the full version of HCL. The distance error is also decreased for all the variants except for $L_{NS},L_{CT}$ that obtains equivalent performance. The best error distance is achieved by $L_{NS},L_{HT}$, but as before, if we consider the variability of the results, the differences w.r.t. the full version of HCL are not significant.

A remarkable outcome we can draw from these experiments is that the Hierarchical Triplets term $L_{HT}$ improves the average metrics at the cost of increasing the variability of the method. This is expected as this constraint introduces different information that depends on the training data. As discussed in Section 3.2, we make triplets from the batch that is fed to the network. If batches are different, so will the triplets also be. In the case of MTSD, which is a much larger dataset than TT100K, it is possible to make a much larger number of triplets that introduce different information, consequently affecting the training result. This is also applied to the C-triplet term $L_{CT}$ for the same reason. There are more available pairs of different classes in a larger dataset, therefore affecting the training outcome.

It is also worth mentioning that the variability of $L_{HC}$ is expected to be low due to the kind of experiments we carry. Its cost depends on the angle between class *prototypes*. Using fixed pre-computed features that are not changing through the training only requires finding the class *prototypes*. Training the ResNet-101 backbone would also be translated into a higher variability for this term.

In summary, this ablation study shows that the proposed HCL terms can help with improving the performance, as shown in MTSD results. On TT100K, they do not improve the performance of the NSL alone on average because it already reaches a very high value. Some of the HCL terms ($L_{HT},L_{CT}$) have shown to increase the potential performance at the cost of increasing the variability of the results. Triplet mining strategies might help to mitigate this issue.

## 7. Conclusions

We have addressed the problem of hierarchical novelty detection, specifically focused on traffic sign recognition. It involves classification along with detection of novel classes, and consists of predicting not only that a sample belongs to a novel class (never seen during training), but also its closest position in a semantic hierarchy of known classes. We have introduced a novel loss function, Hierarchical Cosine Loss, that learns jointly an embedding of discriminative features consistent with the class taxonomy, as well as *prototype* representations for both leaf and parent classes. HCL achieves equivalent results to state-of-the-art approaches on natural images benchmarks, AWA2 and CUB, and significantly outperforms them on traffic sign datasets. For the latter experiments, we have contributed taxonomies and corresponding training splits for TT100K and MTSD, two challenging large scale traffic signs benchmarks that simulate real data of a traffic sign recognition application. Our approach is able to detect novel samples from unknown classes at the correct nodes of the hierarchy with 75% and 24% accuracy when we classify known classes with 90% accuracy, for TT100K and MTSD, respectively. It also reaches 81% and 36% novel accuracy at 80% known accuracy, for TT100K and MTSD, respectively. Finally, we have contributed an ablation study that analyzes the individual performance of the HCL terms.

As a future line of research, our model could be applied to class incremental learning. By adding the *prototypes* of the detected novel classes at the proper taxonomy locations, our model could be extended to recognize new classes.

## Figures and Tables

**Table 1:** Datasets overview: number of samples, parent and leaf classes in the taxonomy tree and its height, for both known and novel splits. The height of a tree is the height of its root class, so that a tree of two levels is of height 1.

	Known				Novel	
Dataset	# Samples	# Parents	# Leaves	Height	# Classes	# Samples
AWA2	29,408	21	40	5	10	7913
CUB	8814	43	150	5	50	2966
TT100K	21,956	14	80	2	23	1735
MTSD	65,312	40	164	3	39	4743

**Table 2:** Comparison of HCL against *TD+LOO* [5] and *Relabel* [5] on AWA2 and CUB. Performance is measured by the novel/known accuracy AUC and the novel accuracy and average hierarchical error distance ${\bar{d}}_{h}$ at 50% known accuracy. The reported values are the average from a set of 50 experiments ±2σ. Best metrics are highlighted in bold.

	AWA2			CUB		
Method	AUC	Novel acc	Novel ${\bar{d}}_{h}$	AUC	Novel acc	Novel ${\bar{d}}_{h}$
		@50%↑	@50%↓		@50%↑	@50%↓
TD+LOO [5]	25.7 ± 4.3	33.1 ± 6.1	1.82 ± 0.23	18.0 ± 1.0	9.9 ± 1.1	2.56 ± 0.10
Relabel [5]	**33.7 ± 5.9**	**38.7 ± 6.8**	**1.65 ± 0.12**	**28.9 ± 2.2**	**38.1 ± 3.5**	1.56 ± 0.07
HCL	32.8 ± 1.8	36.4 ± 2.2	1.95 ± 0.05	27.6 ± 0.6	35.7 ± 1.2	**1.34 ± 0.03**

**Table 3:** Comparison of HCL against the state-of-the-art models on TT100K and MTSD. Performance is measured by the novel/known accuracy AUC and the novel accuracy and average hierarchical error distance ${\bar{d}}_{h}$ at 50% and 70% known accuracies. The values are the average from a set of 50 experiments ±2σ. Best metrics are highlighted in bold.

Dataset	Method	AUC	Novel acc ↑		Novel ${\bar{d}}_{h}$ ↓	
			@50%	@70%	@50%	@70%
	TD+LOO [5]	42.2 ± 2.6	55.8 ± 4.6	12.6 ± 1.5	0.68 ± 0.09	1.47±0.20
TT100K	Relabel [5]	48.4 ± 3.7	52.3 ± 4.0	48.7 ± 4.2	0.63 ± 0.06	0.66 ± 0.07
	HCL	**84.1 ± 0.7**	**87.2 ± 0.8**	**83.7 ± 1.1**	**0.15 ± 0.01**	**0.18 ± 0.01**
	TD+LOO [5]	30.6 ± 1.5	36.4 ± 2.6	9.5 ± 1.9	1.31 ± 0.10	2.12 ± 0.08
MTSD	Relabel [5]	27.3 ± 3.8	30.9 ± 5.0	24.6 ± 3.6	1.28 ± 0.11	1.44 ± 0.09
	HCL	**44.2 ± 1.6**	**47.7 ± 2.1**	**40.5 ± 2.3**	**0.78 ± 0.04**	**0.89 ± 0.05**

**Table 4:** Comparison of different training strategies for HCL on MTSD and TT100K. I stands for ImageNet features, F for fine-tuned features and B for training both the backbone and HCL simultaneously. We report the novel/known accuracy AUC and the novel accuracy and average hierarchical error distance ${\bar{d}}_{h}$ at 50%, 70%, and 80% known accuracy points. Best metrics are highlighted in bold.

	AUC	Novel acc ${\bar{d}}_{h}$ ↑			Novel ${\bar{d}}_{h}$ ±2σ ↓		
	±2σ	@50%	@70%	@80%	@50%	@70%	@80%
	TT100K						
I	54.0 ± 3.2	63.2 ± 2.8	45.3 ± 4.5	27.4 ± 4.6	0.43 ± 0.03	0.69 ± 0.02	0.95 ± 0.02
F	**84.1 ± 0.7**	**87.2 ± 0.8**	**83.7 ± 1.1**	**80.7 ± 1.0**	**0.15 ± 0.01**	**0.18 ± 0.01**	**0.22 ± 0.01**
B	71.4 ± 4.1	80.0 ± 7.8	60.6 ± 9.3	50.9 ± 6.5	0.22 ± 0.08	0.42 ± 0.09	0.54 ± 0.06
	**MTSD**						
I	25.9 ± 1.0	34.3 ± 1.4	00.0±0.0	00.0±0.0	1.36 ± 0.02	-	-
F	**44.2 ± 1.6**	**47.7 ± 2.1**	**40.5 ± 2.3**	**35.8 ± 2.2**	**0.78 ± 0.04**	**0.89 ± 0.05**	**0.99 ± 0.05**
B	43.1 ± 8.2	47.4 ± 11.3	36.7 ± 10.9	30.8 ± 7.1	1.01 ± 0.20	1.16 ± 0.22	1.26 ± 0.19

**Table 5:** Ablation study of the HCL terms. Performance is measured by the novel/known accuracy AUC and the novel accuracy and average hierarchical error distance ${\bar{d}}_{h}$ at 70% and 80% known accuracies. The metrics are the average of 10 experiments ±2σ. Best metrics are highlighted in bold.

Losses	$\{\lambda_{\mathrm{NS}},\lambda_{\mathrm{HC}},$	AUC	Novel acc ±2σ ↑		Novel ${\bar{d}}_{h}$ ±2σ ↓	
	$\lambda_{\mathrm{CT}},\lambda_{\mathrm{HT}}\}$	±2σ	@70%	@80%	@70%	@80%
		TT100K				
$L_{NS}$	{1,0,0,0}	**84.1 ± 0.6**	**83.9 ± 0.8**	**80.9 ± 0.7**	**0.18 ± 0.01**	**0.21 ± 0.01**
$L_{NS},L_{HC}$	{1,10,0,0}	84.0 ± 0.4	83.7 ± 0.5	80.7 ± 0.7	0.19 ± 0.01	0.22 ± 0.01
$L_{NS},L_{CT}$	{1,0,1,0}	83.8 ± 0.9	83.6 ± 1.3	80.6 ± 1.2	0.19 ± 0.01	0.22 ± 0.01
$L_{NS},L_{HT}$	{1,0,0,0.1}	**84.1 ± 0.7**	83.8 ± 0.9	80.7 ± 0.8	**0.18 ± 0.01**	0.22 ± 0.01
HCL	{1,10,1,0.1}	**84.1 ± 0.7**	83.7 ± 1.1	80.7 ± 1.0	**0.18 ± 0.01**	0.22 ± 0.01
		**MTSD**				
$L_{NS}$	{1,0,0,0}	41.8 ± 0.5	37.6 ± 0.7	33.7 ± 0.7	0.93 ± 0.01	1.03 ± 0.01
$L_{NS},L_{HC}$	{1,10,0,0}	42.8 ± 1.1	38.9 ± 1.4	34.7 ± 1.4	0.91 ± 0.03	1.01 ± 0.02
$L_{NS},L_{CT}$	{1,0,1,0}	42.5 ± 2.2	38.5 ± 2.8	34.1 ± 2.8	0.94 ± 0.07	1.04 ± 0.07
$L_{NS},L_{HT}$	{1,0,0,0.1}	43.7 ± 1.2	39.9 ± 1.7	35.7 ± 1.8	**0.88 ± 0.03**	**0.97 ± 0.03**
HCL	{1,10,1,0.1}	**44.2 ± 1.6**	**40.5 ± 2.3**	**35.8 ± 2.2**	0.89 ± 0.05	0.99 ± 0.05

**Figure A1:**
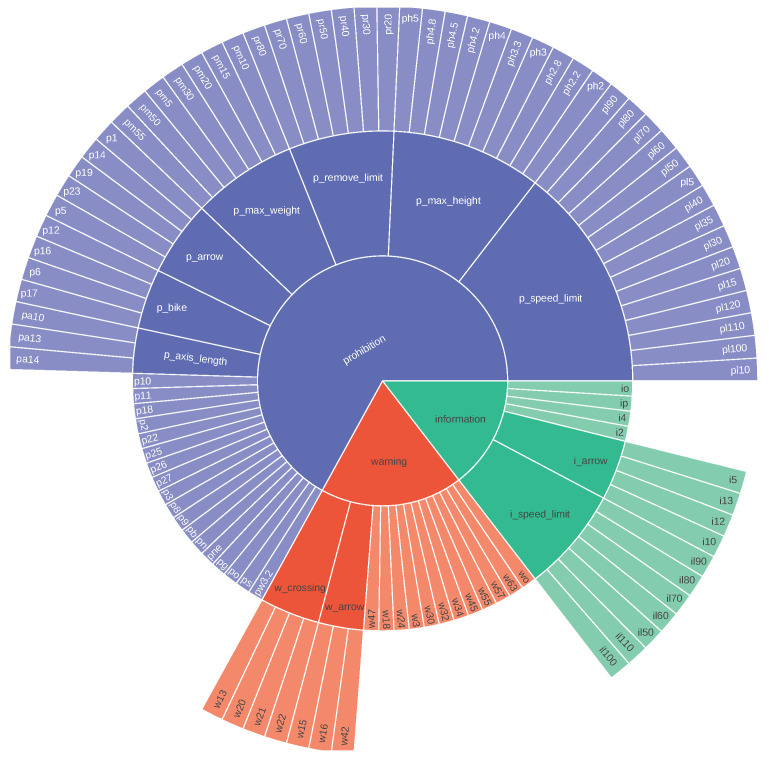
TT100K class taxonomy. It contains both novel and known classes. See at full size in [36].

**Figure A2:**
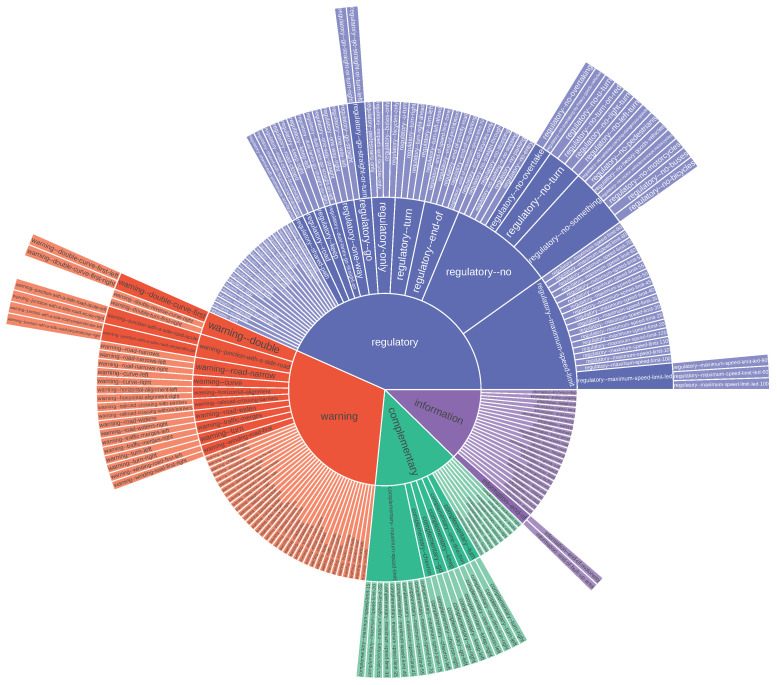
MTSD class taxonomy. It contains both novel and known classes. See at full size in [36].

**Figure A3:**
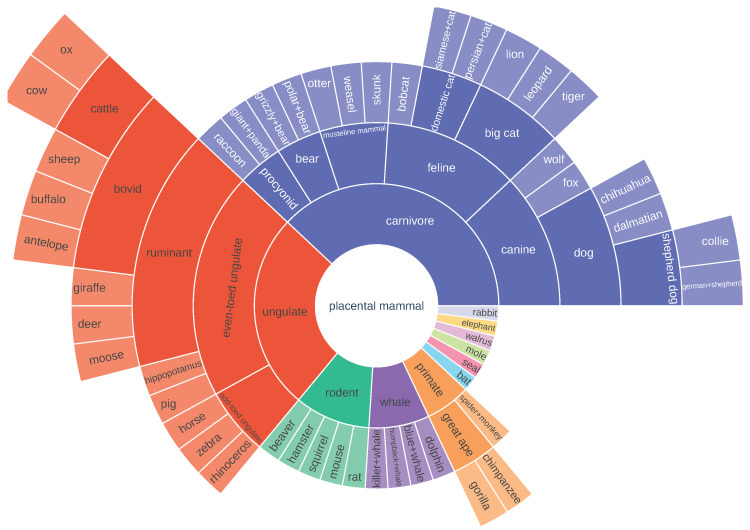
AWA2 class taxonomy. It contains both novel and known classes. See at full size in [36].

**Figure A4:**
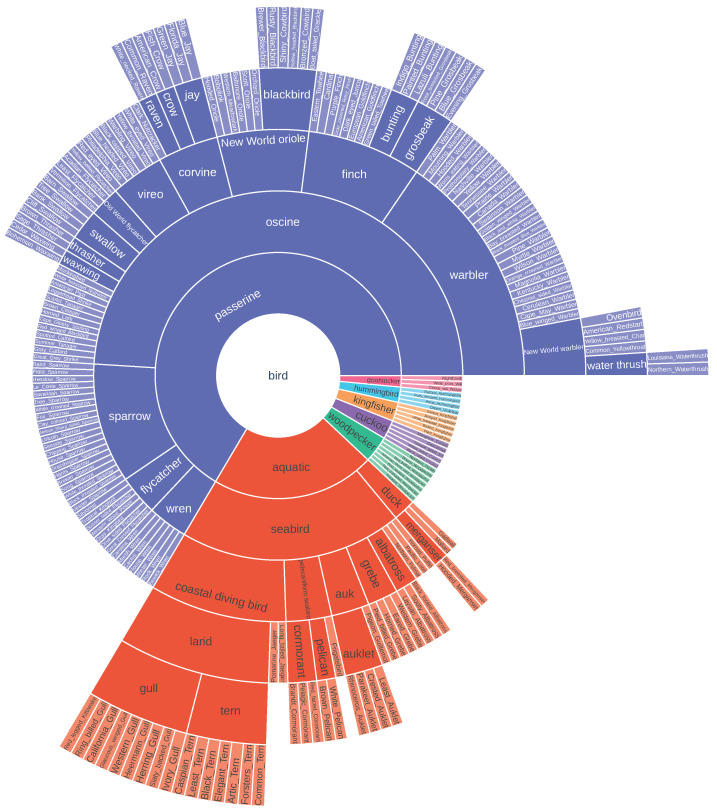
CUB class taxonomy. It contains both novel and known classes. See at full size in [36].

## Data Availability

The necessary data to reproduce the experiments as well as the implementation of the method proposed in this manuscript are available in a GitHub public repository [36].

## Abbreviations

The following abbreviations are used in this manuscript:

HCL	Hierarchical Cosine Loss
NSL	Normalized Softmax Loss
MTSD	Mapillary Traffic Sign Dataset
TT100K	Tsinghua-Tencent 100K

## Appendix A. Hyperparameters

All the hyperparameters were tuned by optimizing them to the validation set. Note that, in hierarchical novelty detection, there is no split of novel samples used as validation to search for hyperparameters. This, as discussed in [5], makes the problem more challenging, as we can only optimize our model to the validation set of the known classes, but the novel classes will always remain unknown.

### Appendix A.1. State-of-the-Art Models

For the experiments on CUB and AWA, we use the parameters provided by the authors [5]. They train in a full-batch manner using an Adam optimizer with an initial learning rate of $10^{-2}$, and it decays at most two times when loss improvement is less than 2 compared to the last epoch. They apply L2 norm weight decay with parameter $10^{-2}$.

For MTSD and TT100K, we keep the same setting, except for the relabeling rate of the Relabel model that was set to 15% and 30%, respectively.

### Appendix A.2. HCL

For all the experiments on HCL, we use an Adam optimizer with a learning rate of 0.01 and the hyperparameters from Table A1. In the experiments where we jointly train the backbone and HCL, the Adam optimizer uses a learning rate of $10^{-4}$ for the ResNet-101 backbone, for both datasets. Table A1 contains: the regularization parameters for the HCL loss, $\left\{\lambda_{NS},\lambda_{HC},\lambda_{CT},\lambda_{HT}\right\}$, the batch size (BS), number of epochs ($n_{epochs}$) and relabeling rate $r_{rate}$. On the experiments of HCL being trained on precomputed features, we employ a full-batch training. The *s* parameter from the Normalized Softmax loss is always set to 40.

For the experiments in the ablation study, we keep the same hyperparameters as when we train the full version of HCL.

**Table A1:** Hyperparameters to train HCL.

Experiment	$\left\{\lambda_{\mathrm{NS}},\lambda_{\mathrm{HC}},\lambda_{\mathrm{CT}},\lambda_{\mathrm{HT}}\right\}$	BS	$n_{\mathrm{epochs}}$	$r_{\mathrm{rate}}\left(\%\right)$
AWA2				
Imagenet feat.	{1,1,1,0.01}	full-batch	1000	15
CUB				
Imagenet feat.	{1,1,1,0.01}	full-batch	1000	15
TT100K				
Imagenet feat.	{1,1,1,0.01}	full-batch	1000	30
Finetuned feat.	{1,10,1,0.1}	full-batch	1000	30
Backbone + HCL	{1,10,1,10}	280	300	30
MTSD				
Imagenet feat.	{1,1,1,0.01}	full-batch	1000	15
Finetuned feat.	{1,10,1,0.1}	full-batch	1000	15
Backbone + HCL	{1,10,1,10}	280	300	15

## Appendix B. Taxonomy Figures

Taxonomies for TT100K, MTSD, AWA2, and CUB are depicted in Figure A1, Figure A2, Figure A3 and Figure A4, respectively.
